# Influence of deprivation on initial severity and prognosis of patients admitted to the ICU: the prospective, multicentre, observational IVOIRE cohort study

**DOI:** 10.1186/s13613-020-0637-1

**Published:** 2020-02-11

**Authors:** Jean-Pierre Quenot, Julie Helms, Guylaine Labro, Auguste Dargent, Nicolas Meunier-Beillard, Elea Ksiazek, Pierre-Edouard Bollaert, Guillaume Louis, Audrey Large, Pascal Andreu, Christophe Bein, Jean-Philippe Rigaud, Pierre Perez, Raphaël Clere-Jehl, Hamid Merdji, Hervé Devilliers, Christine Binquet, Ferhat Meziani, Isabelle Fournel, the IVOIRE Trial Investigators and the CRICS TRIGGERSEP Group (Clinical Research in Intensive Care and Sepsis Trial Group for Global Evaluation and Research in Sepsis), Bruno Lévy, Jérémie Lemarié, Cyril Cadoz, Antoine Marchalot, Alexandra Monnier, Yannick Rabouel

**Affiliations:** 1 Service de Médecine Intensive-Réanimation, CHU Dijon Bourgogne, 14 rue Paul Gaffarel, B.P 77908, 21079 Dijon Cedex, France; 2 INSERM, U1231, Equipe Lipness, Dijon, France; 3 LipSTIC LabEx, Fondation de coopération scientifique Bourgogne-Franche-Comté, Dijon, France; 4 INSERM, CIC 1432, Module Epidémiologie Clinique, Dijon, France; 5 Hôpitaux universitaires de Strasbourg, Service de Réanimation, Nouvel Hôpital Civil, Strasbourg, France; 6 Université de Strasbourg (UNISTRA), Faculté de Médecine, Strasbourg, France; 7 Service de Réanimation Médicale, CHU de Besançon, Besançon, France; 8 DRCI, USMR, CHU Dijon Bourgogne, Dijon, France; 9 Service de Médecine Intensive-Réanimation, CHRU Central, Nancy, France; 10 Service de Réanimation Médicale, CH de Metz, Metz, France; 11 Service de Réanimation Polyvalente, CH de la Haute-Saône, Vesoul, France; 12 Service de Médecine Intensive Réanimation, CH de Dieppe, Dieppe, France; 13 Service de Réanimation Médicale, CHRU Brabois, Nancy, France; 14 Service de Médecine Interne et Maladies Systémiques, CHU Dijon Bourgogne, Dijon, France; 15 INSERM (French National Institute of Health and Medical Research), UMR 1260, Regenerative Nanomedicine (RNM), FMTS, Strasbourg, France

**Keywords:** Socioeconomic, Deprivation, Critically ill, Intensive care unit

## Abstract

**Background:**

The influence of socioeconomic status on patient outcomes is unclear. We assessed the impact of socioeconomic deprivation on severity of illness at intensive care unit (ICU) admission, and on the risk of death at 3 months after ICU admission.

**Methods:**

The IVOIRE study was a prospective, observational, multicentre cohort study in the ICU of 8 participating hospitals in France, including patients aged ≥ 18 years admitted to the ICU and receiving at least one life support therapy for organ failure. The primary outcomes were severity at admission (assessed by SAPSII score), and mortality at 3 months. Socioeconomic data were obtained from interviews with patients or family. Deprivation was assessed using the EPICES score.

**Results:**

Among 1294 patents included between 2013 and 2016, 629 (48.6%) were classed as deprived and differed significantly from non-deprived subjects in terms of sociodemographic characteristics and pre-existing conditions. The mean SAPS II score at admission was 50.1 ± 19.4 in deprived patients and 52.3 ± 17.3 in non-deprived patients, with no significant difference by multivariable analysis (*β* = − 1.85 [95% CI − 3.86; + 0.16, *p* = 0.072]). The proportion of death was 31.1% at 3 months, without significant differences between deprived and non-deprived patients, even after adjustment for confounders.

**Conclusions:**

Deprivation is frequent in patients admitted to the ICU and is not associated with disease severity at admission, or with mortality at 3 months between deprived and non-deprived patients.

*Trial registration* The IVOIRE cohort is registered with ClinicalTrials.gov under the identifier NCT01907581, registration date 17/7/2013

## Background

Despite the outstanding progress achieved in critical care in recent years, mortality remains high among patients admitted to the intensive care unit (ICU) [1, 2]. Mortality in ICU patients reflects the severity of the disease justifying the ICU admission, which is often assessed using scores such as the SAPS II, APACHE II or SOFA [3–5]. However, although these scores are relevant to compare the profiles of patients across ICUs, or as adjustment variables in clinical trials, they are no longer the only parameters that should be taken into account to explain mortality in the ICU. While the severity of disease leading to admission naturally accounts for a large part in explaining mortality, other aspects also need to be taken into consideration.

Socioeconomic status, psychosocial, material and behavioural factors are among the many parameters that contribute to inequalities in healthcare. These factors can compound pre-existing vulnerability, or render vulnerable patients who are already weakened by underlying illness [6–8]. The impact of health inequalities was investigated in a large study in 22 countries across all parts of Europe, based on hospital admission registers. The authors reported that the rates of death were significantly higher in groups with lower socioeconomic status, as assessed by level of education, occupational class and income [9]. They also found that substantial inequalities in health existed between higher and lower socioeconomic groups, although the magnitude of the difference varied across countries [9]. These findings have also been confirmed in other specific clinical contexts, such as trauma, cardiovascular disease and cancer [10–14].

In the field of critical care, a number of studies have investigated the influence of socioeconomic status on patient outcomes [15–20]. However, they have all been the object of some criticism, notably because they were single-centre, used retrospective or medical informatics data, had short follow-up (up to ICU or hospital discharge only), analysed subgroups only, or because other factors influencing the prognosis of patients in ICU and after discharge were not taken into account. In addition, previous studies specifically in France investigating the relation between socioeconomic status and outcomes included different patient populations (e.g. homeless subjects [21]) or used different methods to evaluate deprivation [21, 22].

In this context, the present study was designed to assess the impact of deprivation on severity of illness at ICU admission, and on mortality at 3 months after ICU admission.

## Methods

### Design and study population

The IVOIRE study was a prospective, observational, multicentre cohort study in patients admitted to the ICU in 8 participating centres [5 university teaching hospitals and 3 mixed ICUs from general (non-academic) hospitals].

All patients aged ≥ 18 years admitted to the ICU and receiving at least one life support therapy for organ failure (mechanical ventilation, vasopressors or inotropic agents, renal replacement therapy, high flow nasal cannula) were eligible for inclusion. Patients were only included once (i.e. not included a second time in case of re-admission). Patients who were capable of being interviewed, either personally or via a next of kin were included. Patients with major cognitive impairment before admission to the ICU, patients for whom follow-up at 3 months was anticipated to be impossible (e.g. homeless persons), patients with no social security coverage, and patients under legal guardianship or judicial protection were not included.

The study received approval for all participating centres from the local Ethics Committee (Comité de Protection des Personnes Est I) under the number 2013/15 and from the French national agency for the safety of medical products and devices (Agence National de Sécurité des Médicaments et des Produits de Santé, ANSM, approval number 121506B-31). The study was registered with ClinicalTrials.gov under the identifier NCT01907581. All patients and/or their next of kin were informed and consent was documented in the patients’ medical records by the investigator.

This study is reported in compliance with the STROBE guidelines [23, 24].

### Outcomes

The primary outcomes were severity at admission, as assessed by the SAPS II score at ICU admission [3], and mortality at 3 months following admission. The SAPS II score was considered as a continuous variable. Secondary outcomes were mortality recorded at 6 and 12 months. For patients who could not be contacted at follow-up, a sequential procedure was followed, namely: telephone contact with the patient, or their family; telephone contact with the general practitioner (GP), or other specialists, then finally, vital status was obtained from the national death registry.

### Variables of interest and data collection

Deprivation status was obtained from interviews with the patients or their next-of-kin during the ICU stay using the EPICES (Evaluation de la Précarité et des Inégalités de santé dans les Centres d’Examen de santé, Evaluation of Deprivation and Inequalities in Health Examination Centres) score (Additional file 1). The EPICES score [25] measures social and material deprivation and is based on a multidimensional questionnaire composed of 11 items relating to social conditions, leisure activities and family/social support. This score ranges from 0 to 100, from the lowest to the highest level of deprivation, but it is mostly used as a dichotomous variable to discriminate deprived and non-deprived patients. Patients with an EPICES score ≥ 30.17 (lower boundary of the fourth quintile in the validation study) were considered as socially deprived [25–27]. This score, validated in a large French cohort of 197,389 patients, has been shown to be a reliable index to measure social deprivation at an individual level [25, 28].

Other data collected included living conditions (own home, retirement home, nursing home), level of education and professional qualification, occupational position, healthcare coverage. We also recorded demographic characteristics, reasons for ICU admission, comorbidities as evaluated by the Charlson index [29], smoking status, level of alcohol intake, Katz’s Activities of Daily Living (ADL) [30], variables relating to healthcare utilization (chronic disease, home help, time required to get to the nearest doctor). Severity of disease at baseline was calculated using the Simplified Acute Physiological Score (SAPS) II [3] and Sequential Organ Failure Assessment (SOFA) score [31] at ICU admission. Life support therapy in ICU (mechanical ventilation, vasopressors and/or inotropic agent, renal replacement therapy, high flow nasal cannula), length of ICU and hospital stay were also collected.

Dedicated clinical research assistants collected all data using a standardized electronic case report form. Automatic checks were generated for missing or incoherent data.

We also recorded for each patient the level of social security coverage. France offers universal coverage for all citizens and legal residents, regardless of age or economic situation. Citizens and residents are covered through mandatory health insurance contributions up to approximately 70 to 75% of costs, and optional private insurance is available for those who want additional coverage (to bring coverage up to 100%). Free complementary health insurance is provided for permanent residents of France whose household income falls below a certain threshold. State medical assistance is made available for foreigners whose application for legal residence has not yet been finalized. State medical assistance is provided below a certain level of income, and covers up to 100% of healthcare costs (100% of the tariffs determined by the social welfare system).

### Sample size calculation

The mean SAPS II in the ICU of the coordinating centre was 55 (estimates derived from internal statistics). We calculated that a sample size of 1400 participants would be needed to detect an increase in predicted mortality of 10%, which corresponds to a SAPS II score of 60, and assuming that 20% of our population [32] would be classified as deprived at an alpha risk of 5% and power of 80%. This sample size would also enable us to detect OR varying from 1.44 to 1.52 according to deprivation frequency, and assuming expected mortality of 40% in non-deprived patients.

### Statistical analysis

Continuous variables are expressed as mean ± standard deviation, or median [Q1, Q3], and categorical variables as number (percentage). Group comparisons were performed using the Chi-square or Fisher’s exact test for categorical variables, Student’s *t* test for normally distributed data and the Wilcoxon rank sum test otherwise.

The impact of deprivation status on the severity of illness at ICU admission was assessed by a linear regression model and adjusted for clinically relevant factors (age, sex, level of education, Charlson comorbidity index and ADL score), and centre.

The impact of deprivation status on mortality at 3, 6 and 12 months was studied with a logistic regression model adjusted for clinically relevant prognostic factors (age, SAPS II, ADL score, Charlson comorbidity index) and centre.

Analyses were performed on the complete-case population, and sensitivity analyses were also performed on the imputed population to account for any patients with missing data. Multiple imputations by the chained equation method were performed using 10 imputed datasets. The variables used for imputation were age, sex, SAPS II, Charlson comorbidity index, mortality at 3, 6 and 12 months, the EPICES score, the ADL score, presence of chronic disease, level of education and centre.

The association between deprivation status and life support therapy was also assessed using the Chi-square test, and then by multivariate logistic regression adjusted for SAPS II, septic shock, cardiogenic shock and centre, based on clinical relevance. The association between deprivation status and the destination after discharge (home/institution/hospital) was assessed by multivariate multinomial logistic regression.

All analyses were performed using SAS version 9.4 (SAS Institute Inc., Cary, NC, USA) with a significance threshold of less than 0.05 in 2-sided tests.

## Results

**Fig. 1:**
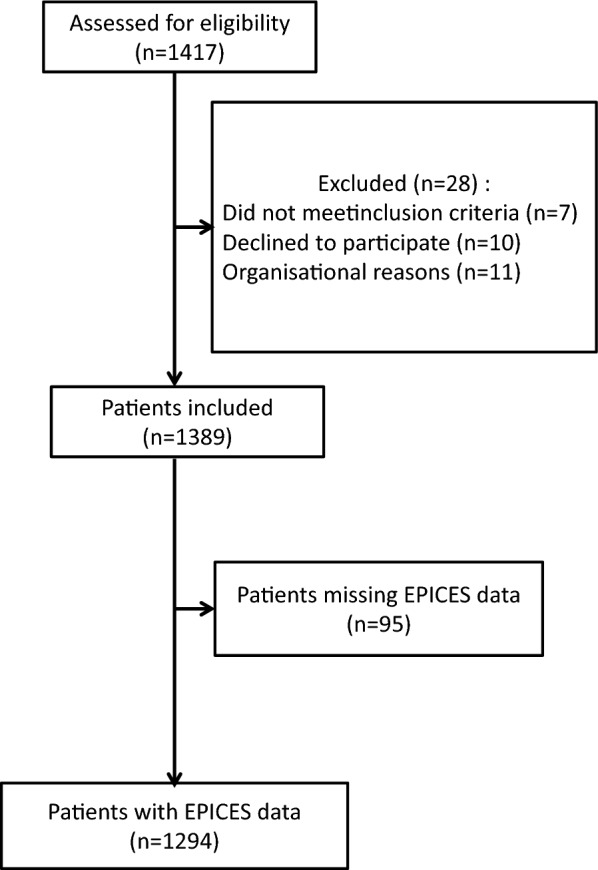
Flowchart of the IVOIRE study population

From 06 June 2013 to 22 January 2016, a total of 1417 patients were screened for eligibility. Among the 1389 patients who consented to participate in the IVOIRE study and who met the inclusion criteria, 95 patients were excluded from the present analysis due to missing information regarding EPICES score. A flowchart of the study population is shown in Fig. 1. The characteristics of the 1294 patients included in the final analysis are presented in Table 1. Among these, 629 (48.6%) were classed as deprived. EPICES score ranged from 0 to 29.6 in non-deprived patients (mean = 17.2 ± 8.8), and from 30.17 to 100 in deprived patients (mean = 47.6 ± 13.8). Deprived patients differed significantly from non-deprived subjects in terms of sociodemographic characteristics (younger, lower level of education, more often on invalidity or without professional activity), living arrangements (more often living alone) and pre-existing conditions (higher alcohol and tobacco consumption, and dependency). Patients who were deprived more frequently had chronic disease or home help, had greater difficulty paying for their medication and medical exams, and needed more time to get to the nearest GP. Basic French healthcare coverage and complementary health insurance were also less common in deprived patients, contrary to universal coverage and state medical aid, which were more frequent in these patients. Deprived patients more frequently consulted GPs or specialists in the year prior to their ICU admission, and more frequently had a history of at least one hospital admission in the last year, but less frequently consulted the dentist as compared to non-deprived patients (Additional file 2: Table S1).

**Table 1:** Comparison of deprived and non-deprived patients at admission to the intensive care unit in the IVOIRE cohort (*N* = 1294)

Variable	All (*n* = 1294)	Non-deprived (*n* = 665)	Deprived (*n* = 629)	*p*
Sociodemographic characteristics				
Age	66.17 ± 14.93	67.18 ± 13.95	65.10 ± 15.84	0.01
Male gender	805 (62.2%)	425 (63.9%)	380 (60.4%)	0.195
Level of education^a,c^				< 0.0001
No educational diplomas/certificates	249 (19.7%)	95 (14.6%)	154 (25.0%)	
Primary school certificate and/or some secondary school	363 (28.7%)	173 (26.6%)	190 (30.8%)	
Professional certificate	358 (28.3%)	193 (29.7%)	165 (26.8%)	
High school diploma or higher	296 (23.4%)	189 (29.1%)	107 (17.4%)	
Employment status^a,c^				< 0.0001
Not working, unemployed or job-seeker, never worked	143 (11.2%)	48 (7.3%)	95 (15.3%)	
Employed	190 (14.9%)	118 (18.0%)	72 (11.6%)	
Retired	817 (64.1%)	456 (69.7%)	361 (58.1%)	
Invalidity	125 (9.8%)	32 (4.9%)	93 (15.0%)	
Socioeconomic category^a,c^				< 0.0001
Farmers	54 (4.2%)	34 (5.1%)	20 (3.2%)	
Self-employed/own business	106 (8.2%)	59 (8.9%)	47 (7.5%)	
Upper management and liberal professions	130 (10.1%)	95 (14.4%)	35 (5.6%)	
Labourers	397 (30.9%)	165 (25.0%)	232 (37.1%)	
Employees/intermediate professions	500 (38.9%)	273 (41.4%)	227 (36.3%)	
No profession	69 (5.4%)	25 (3.8%)	44 (7.0%)	
Did not wish to disclose	29 (2.3%)	9 (1.4%)	20 (3.2%)	
Living arrangements^b^				
Living alone	372 (28.8%)	105 (15.8%)	267 (42.4%)	< 0.0001
Living with family members	871 (67.3%)	556 (83.6%)	315 (50.1%)	< 0.0001
In assisted living, long-term care facility or nursing home	47 (3.6%)	2 (0.3%)	45 (7.1%)	< 0.0001
Social welfare coverage^b^				
Beneficiary of the basic French healthcare system^c^	1234 (96.4%)	642 (97.9%)	592 (94.9%)	0.004
Universal healthcare coverage^c^	46 (3.6%)	15 (2.3%)	31 (5.0%)	0.010
Universal coverage + assistance with complementary health insurance + state medical aid^c^	47 (3.7%)	11 (1.7%)	36 (5.8%)	0.0001
Complementary health insurance^c^	1102 (86.2%)	612 (93.3%)	490 (78.8%)	< 0.0001
Pre-existing conditions				
Alcohol consumption (≥ 2 glasses per day)^c^	399 (31.7%)	179 (27.5%)	220 (36.1%)	0.001
Smoking status^c^				< 0.0001
Non-smokers	474 (36.8%)	272 (41.0%)	202 (32.4%)	
Current smokers	360 (28.0%)	144 (21.7%)	216 (34.7%)	
Former smokers	453 (35.2%)	248 (37.3%)	205 (32.9%)	
Charlson comorbidity index mean ± SD	2.6 ± 2.5	2.4 ± 2.4	2.8 ± 2.6	0.007
Katz ADL score < 3^c^	88 (6.9%)	30 (4.6%)	58 (9.3%)	0.0009
Healthcare utilization				
Chronic disease^c^	839 (68.0%)	416 (64.8%)	423 (71.4%)	0.0123
Home help^c^	271 (21.4%)	121 (18.4%)	150 (24.5%)	0.009
Difficulty paying for medication or examinations^c^	90 (7.2%)	16 (2.5%)	74 (12.3%)	< 0.0001
Time from home to nearest doctor ≥ 15 min^c^	303 (24.4%)	131 (20.3%)	172 (28.7%)	0.0006
Severity of illness at admission				
SOFA^c^ mean ± SD	8.0 ± 4.0	8.2 ± 3.8	7.7 ± 4.2	0.018
Indication for admission to ICU^a,c^				0.194
Cardiac	188 (15.3%)	104 (16.5%)	84 (14.0%)	
Gastro-enterology	63 (5.1%)	37 (5.9%)	26 (4.3%)	
Neurological	56 (4.6%)	26 (4.1%)	30 (5.0%)	
Renal	40 (3.2%)	17 (2.7%)	23 (3.8%)	
Respiratory	392 (31.8%)	187 (29.6%)	205 (34.1%)	
Sepsis	325 (26.4%)	170 (26.9%)	155 (25.8%)	
Trauma	72 (5.8%)	33 (5.2%)	39 (6.5%)	
Other reasons	96 (7.8%)	57 (9.0%)	39 (6.5%)	

^a^Percentages may not total 100 because of rounding

^b^Percentages may not total 100 because more than one answer was possible

^c^Missing data: level of education(*n* = 28), employment status (*n* = 19), socioeconomic category (*n* = 9), basic healthcare coverage (*n* = 14), universal coverage (*n* = 29), universal coverage + assistance (*n* = 31), complementary insurance (*n* = 16), alcohol (*n* = 35), smoking (*n* = 7), Katz’s ADL score (*n* = 17), long-term disease (*n* = 60), home help (*n* = 25), difficulty paying for medication or examination (*n* = 48), time to nearest doctor (*n* = 51), SOFA(*n* = 8), indication for admission to ICU (*n* = 62)

### Impact of deprivation status on disease severity at admission

There was no significant difference in the indications for admission according to deprivation (Table 1). The mean SAPS II score at admission was lower in deprived patients compared to non-deprived patients (50.1 ± 19.4 versus 52.3 ± 17.3, respectively, *p* = 0.029). This difference was no longer significant by multivariable analysis (adjusted beta = − 1.85 [95% CI − 3.86; + 0.165], *p* = 0.072). Sensitivity analysis including all patients with missing data yielded similar results (Additional file 2: Table S2).

### Management in the ICU

There were no differences in management between deprived and non-deprived patients, except for the use of mechanical ventilation and vasopressors, both significantly less frequent in deprived patients (Additional file 2: Table S3). However, these associations were no longer significant in multivariable logistic regression after adjustment for severity at admission and presence of septic or cardiogenic shock. There was no difference in treatment withholding or withdrawal according to deprivation, regardless of the severity at admission (Additional file 2: Table S3).

### Outcomes after discharge from ICU

There was no significant difference in length of stay in the ICU or in-hospital, or in discharge modalities from the ICU or the hospital according to deprivation status (Table 2). Among patients who were not resident in a nursing home at the time of admission, deprivation was significantly associated with an increased risk of entry into a nursing home at 3 months after discharge (OR = 2.69; 95% CI 1.40–5.17; *p* = 0.004) and at 6 months (OR = 6.06; 95% CI 2.23–16.43; *p* = 0.0004), after adjustment for age and dependency level by multivariable multinomial logistic analysis.

**Table 2:** Outcomes after discharge from the ICU according to deprivation status in the IVOIRE cohort

	Non-deprived (*n* = 665)	Deprived (*n* = 629)	*p*
Length of stay, days (median [IQR])			
ICU	6 [3–11]	5 [3–11]	0.557^b^
In hospital^a^	17 [10–31]	18 [9–35]	0.576^b^
Outcome at ICU discharge, *n* (%)			0.557^c^
Death	128 (19.2%)	118 (18.8%)	
Transfer	530 (79.7%)	500 (79.5%)	
Discharged to home	7 (1.1%)	11 (1.8%)	
Outcome at hospital discharge^a^, *n* (%)			0.987^c^
Death	41 (7.6%)	39 (7.6%)	
Transfer	218 (40.6%)	205 (40.1%)	
Discharged to home	278 (51.8%)	267 (52.2%)	

^a^Among patients alive at ICU discharge (*n* = 1048)

^b^Wilcoxon rank test

^c^Chi-square test

**Fig. 2:**
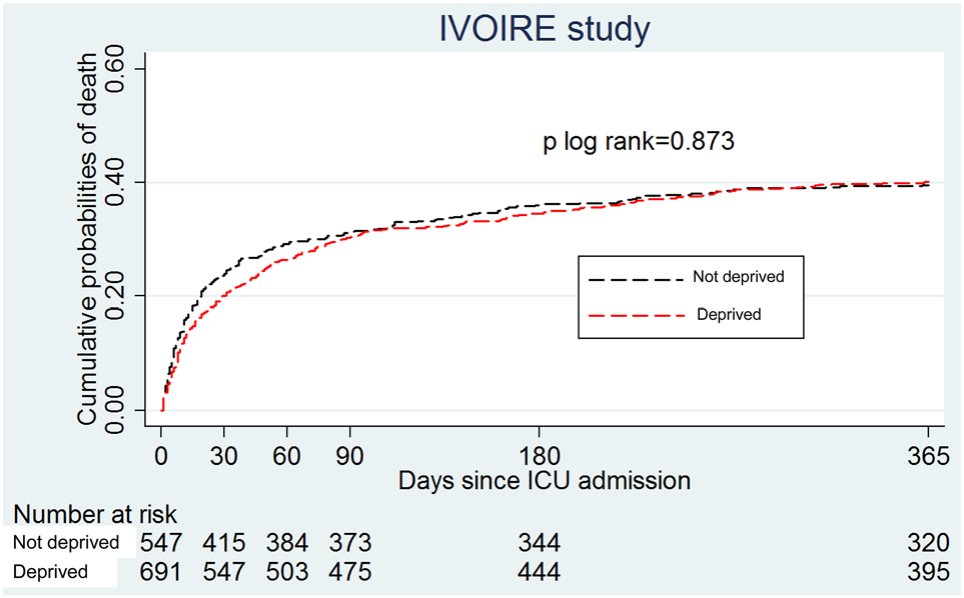
Cumulative probabilities of death within 365 days following ICU admission. *ICU* intensive care unit

The proportion of death was 31.1% at 3 months, 35.3% at 6 months and 40.0% at 12 months, without significant differences between deprived and non-deprived patients, even after adjustment for confounding factors (Table 3; Fig. 2). There was no significant impact of social deprivation on mortality, whatever the score used to assess social vulnerability. Sensitivity analysis including all patients with missing data yielded similar results (Additional file 2: Table S4).

**Table 3:** Impact of deprivation on mortality at 3, 6 and 12 months following ICU admission

	3 months		6 months		12 months	
	Non-deprived	Deprived	Non-deprived	Deprived	Non-deprived	Deprived
Mortality	30.5%	31.6%	35.3%	35.3%	39.1%	40.9%
Crude OR [95% CI] (*n* = 1294)	1	1.05 [0.83; 1.33]	1	1.00 [0.80–1.25]	1	1.07 [0.86; 1.34]
Adjusted OR^a^ [95% CI] (*n* = 1277)	1	1.04 [0.79–1.37]	1	0.97 [0.75–1.27]	1	1.06 [0.82–1.37]

*CI* confidence interval

^a^Multivariate analysis was adjusted for age, SAPS II score, Katz’s ADL, Charlson comorbidity index and centre

## Discussion

This is one of the first studies to be performed in France investigating the impact of deprivation at individual level on severity at admission and mortality. In France, there exist substantial inequalities in healthcare [33, 34], and the healthcare system is profoundly different to the systems used elsewhere in Europe, especially in Anglo-Saxon countries [35, 36]. The French insurance system provides a basic level of medical coverage to all citizens, and 100% coverage for serious or chronic diseases. Our study shows that among all patients admitted to the ICU, just under 50% were deprived, and these patients had similarly severe disease at admission, as assessed by SAPS II and SOFA scores, and no significant difference in mortality at 3 months according to deprivation status. We also observed that deprived patients who were living at home at the time of admission had a higher risk of entry into a nursing home at 3 and 6 months after discharge from the ICU by multivariable analysis. This could be explained by the fact that deprived patients had a higher level of dependence at admission, as assessed by ADLs, and more comorbidities, as assessed by the Charlson index.

The rate of deprived patients admitted to the ICU in our study is difficult to compare with other literature reports, because we used a different definition to classify patients as deprived than previous reports [6–8, 17]. The definition of social deprivation is complex, because it is not a binary state but rather a dynamic concept with different degrees of vulnerability and comprising different dimensions. In our study, we chose to use the EPICES score, which is increasingly implemented in France to evaluate deprivation. Previous studies focused more on healthcare inequalities and socioeconomic factors (including psycho-social and material aspects) that could influence health states in the population, to which may also be added behavioural determinants such as alcohol consumption, smoking, dietary habits, or physical activity [9, 37–41]. A previous study by Bigé et al. among homeless people in France reported that 50% of patients had no health insurance, 56% had no financial resources, and 91% were socially isolated, but that they had the same prognosis overall as housed patients [21]. Our findings are in line with these reports in terms of impact of socioeconomic status on mortality. Conversely, the rate of deprivation observed in our study (48.6%) is higher than that reported in other studies (26% in the study by Bein et al. [17] and 33% in the study by Bastian et al. [22]). These differences may be explained by the fact that these studies included different patient populations, and also used other indices of socioeconomic status. The EPICES score is a multidimensional instrument that encompasses several dimensions and therefore, may be more sensitive.

There are disparities in the reported risk factors for deprivation, and other reports found a much stronger impact of deprivation on prognosis than our study [42–44]. Some authors have previously reported that deprivation was associated with increased severity of patients at admission to the ICU [17, 20, 45], whereas we observed that deprivation was not associated with severity of illness, in line with the findings of others [19]. In deprived patients, we observed a higher level of dependency and more comorbidities at admission, likely explaining why they were admitted to the ICU earlier, and thus, earlier in the course of the disease. Another possible explanation is that deprived patients more frequently consult their GP and specialists, and are therefore more rapidly oriented towards hospitalization as soon as a health problem arises.

Accordingly, they may be more inclined to be hospitalized, especially those with chronic diseases who have 100% healthcare coverage, than non-deprived patients [42, 46]. This phenomenon has previously been observed in a study from the province of Nova Scotia in Canada, which reported that universal healthcare led to significantly greater use of family physician and hospital services among individuals with a lower socioeconomic background [46]. Conversely, we cannot rule out the possibility that the deprived population comprises both patients who are frequent users of the healthcare system, and patients who are far from the healthcare system. This latter group might tend to wait longer before seeking medical help, especially those who live alone [47], likely also with more limited access to intensive care facilities [48, 49]. This is particularly true in areas where there are limited opportunities for intensive care. The combination of insufficient social support, and poorer basic health state with fewer physiological reserves, may lead these patients to be hospitalized from the outset, even though their overall state may be less serious. Few studies to date have investigated the impact of deprivation on medium-term mortality after a stay in the ICU. A recent European, observational cohort study found no association between socioeconomic status and 1-year all-cause mortality after discharge from the ICU [50]. However, in this study, socioeconomic status was not measured at individual level, but was assessed using a composite index that aggregates data based on the zip code of residence to calculate an overall deprivation index. Furthermore, patients who died in the ICU and those with no healthcare coverage were excluded from the study, such that the study population was more highly selected than in the IVOIRE cohort.

We observed that deprived patients had a similar mortality rate at 3 and 6 months, and 1 year as non-deprived patients. The literature can provide clues that might explain this finding. A potential explanation is that patients were managed optimally in the ICU and in post-intensive care, regardless of their deprivation status. The role of allied health professionals such as social workers and rehabilitation services after discharge, as well as home-help organizations likely buffered the potentially deleterious effects of the ICU stay in terms of mortality in the most deprived patients. It is also possible that the frailest and perhaps most deprived patients were not admitted to the ICU because of unfavourable prognosis.

## Study limitations

This study presents some limitations that deserve to be taken into account. Firstly, a small proportion of patients could not be analysed because they were missing data for the EPICES score. However, sensitivity analysis showed that this missing data did not affect the overall results. Secondly, the scale used to evaluate deprivation is validated in France, but the healthcare system in France is quite specific, and therefore results likely can only be extrapolated to countries with health insurance systems of the same type. In addition, we cannot exclude possible interactions between the components of deprivation, or an influence of other unmeasured confounders on deprivation. Indeed, the EPICES score is likely a better reflection of overall vulnerability than actual deprivation in social terms. Thirdly, in the absence of data regarding our target population at the time this study was designed, we hypothesized that 20% of the population would be deprived. In fact, almost 50% were deprived as assessed by the EPICES score. This actually reinforces our findings, by increasing the statistical power of the study. Fourth, the proportion of screened patients who were actually included in the study was low. This probably stems from reasons relating to different durations of participation between centres, different department size and patient profiles, and human resources issues. Indeed, the commitment of some participating centres may have waned or varied between centres, due to the fact that not all centres have dedicated study personnel (such as clinical research assistants, study nurses, etc.). Finally, there may have been other factors that might influence mortality after ICU discharge that were not taken into account, including (but not limited to) rehabilitation, assistance for deprivation, etc.

## Conclusion

Deprivation is frequent in patients admitted to the ICU and is not associated with severity of disease at admission. Although there was no significant difference in mortality at 3 months and 1 year between deprived and non-deprived patients, socially deprived patients more frequently enter a nursing home within the months following ICU discharge. The findings of this study warrant confirmation on a wider scale, with particular focus on the social support provided for deprived patients after discharge from the ICU.

## Supplementary information

**Additional file 1.** EPICES Score.

**Additional file 2: Table S1.** Healthcare utilization according to deprivation status. **Table S2.** Impact of deprivation on SAPS II: β estimates from multivariable linear regression model (1) after multiple imputation procedure and (2) after analysis on complete cases. **Table S3.** Comparison of management in ICU according to deprivation status. **Table S4.** Impact of deprivation on mortality: β estimates from multivariable logistic regression model (1) after multiple imputation procedure and (2) after analysis on complete cases.

## Data Availability

The datasets used and/or analysed during the current study are available from the corresponding author on reasonable request.
