# Supplementary material for: Influence of deprivation on initial severity and prognosis of patients admitted to the ICU: the prospective, multicentre, observational IVOIRE cohort study
Source: Ann Intensive Care. 2020 Feb 11;10:20. doi: 10.1186/s13613-020-0637-1 (PMC7013026; doi:10.1186/s13613-020-0637-1)
Supplement: Supplementary file 1 — Additional file 1. EPICES Score. [file 13613_2020_637_MOESM1_ESM.docx]

**Additional data :** The EPICES score (**E**valuation de la **P**récarité et des **I**négalités de santé dans les **C**entres d’**E**xamens de **S**anté - Evaluation of Deprivation and Inequalities in Health Examination Centres)

| **Questions** | **Score** |  |
| --- | --- | --- |
|  | **Yes** | **No** |
| 1. Do you sometimes meet with a social worker (welfare worker, educator)? | 10.06 | 0 |
| 2. Do you have complementary health insurance? | -11.83 | 0 |
| 3. Do you live maritally? | -8.28 | 0 |
| 4. Are you a homeowner? | -8.28 | 0 |
| 5. Are there periods in the month when you have real financial difficulties in meeting your needs (food, rent, electricity)? | 14.80 | 0 |
| 6. Have you participated in any sports activities in the last 12 months? | -6.51 | 0 |
| 7. Have you gone to any shows (cinema, theatre) in the last 12 months? | -7.10 | 0 |
| 8. Have you gone on holidays during the past 12 months? | -7.10 | 0 |
| 9. Have you seen any family members in the past six months other than your parents or children? | -9.47 | 0 |
| 10. If you were in difficulty (financial, family or health), is there anyone close to you who could provide you with accommodation for a few days? | -9.47 | 0 |
| 11. If you were in difficulty (financial, family or health), is there anyone close to you who could provide you with material assistance? | -7.10 | 0 |
| Intercept | 75.14 | 0 |

The score is calculated by adding the coefficient for question to the intercept whenever the answer is ‘yes’. In our study, patients with an EPICES score above the threshold of 30.17 were considered as deprived.
