# Supplementary material for: Influence of deprivation on initial severity and prognosis of patients admitted to the ICU: the prospective, multicentre, observational IVOIRE cohort study
Source: Ann Intensive Care. 2020 Feb 11;10:20. doi: 10.1186/s13613-020-0637-1 (PMC7013026; doi:10.1186/s13613-020-0637-1)
Supplement: Supplementary file 2 — Additional file 2: Table S1. Healthcare utilization according to deprivation status. Table S2. Impact of deprivation on SAPS II: β estimates from multivariable linear regression model (1) after multiple imputation procedure and (2) after analysis on complete cases. Table S3. Comparison of management in ICU according to deprivation status. Table S4. Impact of deprivation on mortality: β estimates from multivariable logistic regression model (1) after multiple imputation procedure and (2) after analysis on complete cases. [file 13613_2020_637_MOESM2_ESM.docx]

**Table S1:** **Healthcare utilization according to deprivation status**

|  | **All**  **(N=1294)** | **Non deprived**  **(n=665)** | **Deprived**  **(n=629)** | **p** |
| --- | --- | --- | --- | --- |
| **Number of consultations with a general practitioner in the previous 12 months †** |  |  |  | 0.029 |
| ≤ 2 | 231 (18.4%) | 128(19.8%) | 103(17.0%) |  |
| 3-5 | 411 (32.8%) | 227(35.1%) | 184(30.4%) |  |
| > 5 | 611 (48.8%) | 292(45.1%) | 319(52.6%) |  |
| **Number of consultations with a specialist in the previous 12 months †** |  |  |  | 0.013 |
| None | 382 (31.4%) | 184(29.2%) | 198(33.7%) |  |
| 1-2 | 441 (36.2%) | 253 (40.2%) | 188 (32.0%) |  |
| >=3 | 394(32.4%) | 193(30.6%) | 201(34.2%) |  |
| **Number of consultations with a dentist in the previous 12 months †** |  |  |  | <10^-3^ |
| None | 674(56.1%) | 300(47.9%) | 374(64.9%) |  |
| 1 | 356(29.6%) | 219(35.0%) | 137(23.8%) |  |
| >=2 | 172(14.3%) | 107(17.1%) | 65(11.3%) |  |
| **Hospitalized for at least 24 hours during the previous 12 months†** | 610(48.1%) | 287(43.8%) | 323(52.9%) | 0.001 |

**†** Missing data : consultations with general practitioner n=41 ; consultations with specialist n=77 ; consultations with dentist n=92 ; hospitalization in previous 12 months n=27.

**Table S2**: Impact of deprivation on SAPS II: β estimates from multivariable linear regression model 1) after multiple imputation procedure and 2) after analysis on complete cases

|  | Imputed data population (n=1389) | | Complete case population (n=1250) | |
| --- | --- | --- | --- | --- |
|  | Adjusted β * [95% CI] | p | Adjusted β *[95% CI] | p |
| No deprived | Reference |  | Reference |  |
| Deprived | -1.68[—-3.77;+0.42] | 0.12 | -1.85[-3.86; +0.16] | 0.072 |

* Adjusted for age, sex, level of education, Charlson comorbidity index and ADL score and center

**Table S3:** **Comparison of management in ICU according to deprivation status**

|  |  | | | **Bivariable analysis** | | **Multivariable analysis** | |
| --- | --- | --- | --- | --- | --- | --- | --- |
|  | **All (N=1294)** | **Non deprived**  **(n=665)** | **Deprived**  **(n=629)** | **OR crude** | **95% CI** | **ORa** | **95% CI** |
| **Type of Management, n(%)** |  |  |  |  |  |  |  |
| Mechanical ventilation | 937(72.4%) | 508(76.4%) | 429(68.2%) | 0.66 | [0.52-0.85] | 0.79 | [0.60-1.04] |
| High flow nasal cannula **†** | 100(7.7%) | 56(8.4%) | 44(7.0%) | 0.82 | [0.54-1.23] | 0.84 | [0.55-1.29] |
| Vasopressors **†** | 819(63.3%) | 439(66.1%) | 380(60.4%) | 0.78 | [0.62-0.98] | 0.91 | [0.69-1.19] |
| Inotropic agents | 212(16.4%) | 111(16.7%) | 101(16.1%) | 0.96 | [0.71-1.28] | 0.93 | [0.68-1.26] |
| Renal replacement therapy (continuous or intermittent) **†** | 280(21.7%) | 147(22.1%) | 133(21.2%) | 0.95 | [0.73-1.23] | 1.01 | [0.76-1.35] |
| Withholding or withdrawal of treatment | 155(12.0%) | 85 (12.8%) | 70 (11.1%) | 0.85 | [0.61-1.20] | 0.82 | [0.57-1.18] |

**†**Misssing data : High flow nasal cannula (n=1), vasopressors (n=1), renal replacement therapy (n=1)

CPAP, continuous positive airway pressure

*Multivariable analysis was adjusted for SAPS II, septic shock, cardiogenic shock and center. Ajusted OR (Ora) models the probability of each type of management according to deprivation status (reference = no deprivation)

**Table S4**: Impact of deprivation on mortality: OR estimates from multivariable logistic regression model

1) after multiple imputation procedure and 2) after analysis on complete cases

|  | Imputed data population  (n=1389) | | Complete case population  (n=1277) | |
| --- | --- | --- | --- | --- |
|  | Adjusted ORa [95% CI] | P | Adjusted ORa [95% CI] | P |
| **3 months** |  |  |  |  |
| No deprived | Reference |  | Reference |  |
| Deprived | 1.02 [0.89; 1.17] | 0.79 | 1.04 [0.79; 1.37] | 0.80 |
| **6 months** |  |  |  |  |
| No deprived | Reference |  | Reference |  |
| Deprived | 0.99[0.87;1.13] | 0.91 | 0.97 [0.75; 1.27] | 0.84 |
| **12 months** |  |  |  |  |
| No deprived | Reference |  | Reference |  |
| Deprived | 1.04 [0.91; 1.18] | 0.61 | 1.06 [0.82; 1.37] | 0.67 |

Adjusted for age, SAPS II score, Katz’s ADL, Charlson comorbidity index and centre
